# Geometric Catalyst Utilization in Zero-Gap CO_2_ Electrolyzers

**DOI:** 10.1021/acsenergylett.2c02194

**Published:** 2022-11-28

**Authors:** Siddhartha Subramanian, Kailun Yang, Mengran Li, Mark Sassenburg, Maryam Abdinejad, Erdem Irtem, Joost Middelkoop, Thomas Burdyny

**Affiliations:** Materials for Energy Conversion and Storage (MECS), Department of Chemical Engineering, Faculty of Applied Sciences, Delft University of Technology, van der Maasweg 9, 2629 HZ Delft, The Netherlands

## Abstract

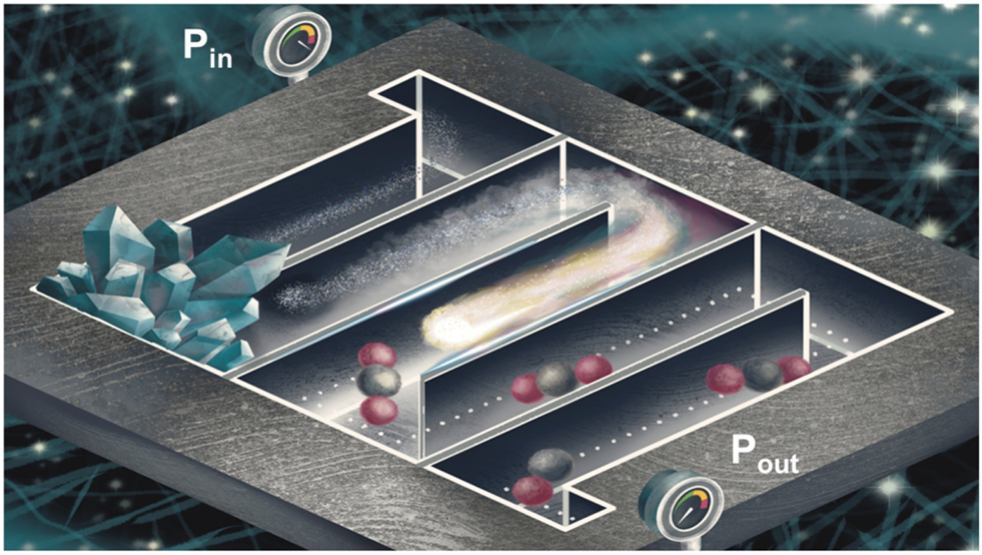

The electrochemical reduction of CO_2_ (CO_2_RR) on silver catalysts has been demonstrated under elevated current density, longer reaction times, and intermittent operation. Maintaining performance requires that CO_2_ can access the entire geometric catalyst area, thus maximizing catalyst utilization. Here we probe the time-dependent factors impacting geometric catalyst utilization for CO_2_RR in a zero-gap membrane electrode assembly. We use three flow fields (serpentine, parallel, and interdigitated) as tools to disambiguate cell behavior. Cathode pressure drop is found to play the most critical role in maintaining catalyst utilization at all time scales by encouraging in-plane CO_2_ transport throughout the gas-diffusion layer (GDL) and around salt and water blockages. The serpentine flow channel with the highest pressure drop is then the most failure-resistant, achieving a CO partial current density of 205 mA/cm^2^ at 2.76 V. These findings are confirmed through selectivity measurements over time, double-layer capacitance measurements to estimate GDL flooding, and transport modeling of the spatial CO_2_ concentration.

The electrochemical CO_2_ reduction reaction (CO_2_RR) is a key enabler to the production of value added chemicals such as CO, ethylene, ethanol, formic acid, and other products.^1−7^ To achieve industrially relevant reaction rates (>100 mA/cm^2^) and lower costs versus alternate production routes, CO_2_RR using gas-diffusion electrodes (GDEs) in a membrane electrode assembly (MEA) configuration is an attractive option due to their reduced cell voltages.^8−14^

In a MEA configuration for CO_2_RR, anion exchange membranes (AEMs) are commonly adopted as the anode and cathode separator, as this maintains an alkaline environment at the cathode, which is more favorable for CO_2_RR selectivity than acidic media. Such a configuration is inherently unstable, however, as excess acidic CO_2_ is continually fed into the reaction environment, leading to two operational challenges. First, the loss of reactant CO_2_ due to its reaction with electrogenerated hydroxide (OH^–^) ions decreases CO_2_ utilization significantly.^15^ Second, due to low liquid volumes and high ion concentrations in the pores of the cathode in an MEA configuration, (bi)carbonate salts are highly prone to precipitate in the cathode catalyst layer, gas-diffusion layer, and CO_2_ gas channel.^16^ Salt deposits have been shown to block access of CO_2_ to catalytic sites while accelerating GDE flooding, which further reduces the amount of CO_2_ reaching the catalyst. Each of these aspects causes the spatial catalyst utilization of a planar electrode area to be decreased during operation, resulting in the competing hydrogen evolution reaction (HER) to replace CO_2_RR in these regions and an overall lower CO_2_RR Faradaic efficiency being measured.^17,18^

Additionally, as the geometric area of electrolyzers increases and higher single-pass conversion efficiencies are targeted,^19−21^ a spatial variation in reactant distribution will also be present along the gas channel of a reactor as reactant is consumed. Importantly, since the area of the gas channel in contact with the GDL is much less than the geometric catalyst area, gas must also be transported in-plane through the GDL to reach catalytic sites adjacent to the current collector (see Figure 1a). Such transport can occur through both diffusion and under-rib convection of CO_2_, which is heavily influenced by the flow field design that is used. Without proper consideration of transport from the gas channel to the immersed catalyst layer, some areas of the catalyst layer may be depleted of CO_2_ even if ample CO_2_ is still present in the gas channel. In brief, there are a multitude of factors which then affect the ability for CO_2_ to reach all geometric areas of a CO_2_ electrolyzer and for the catalyst to be fully “utilized” for CO_2_RR.

**Figure 1:**
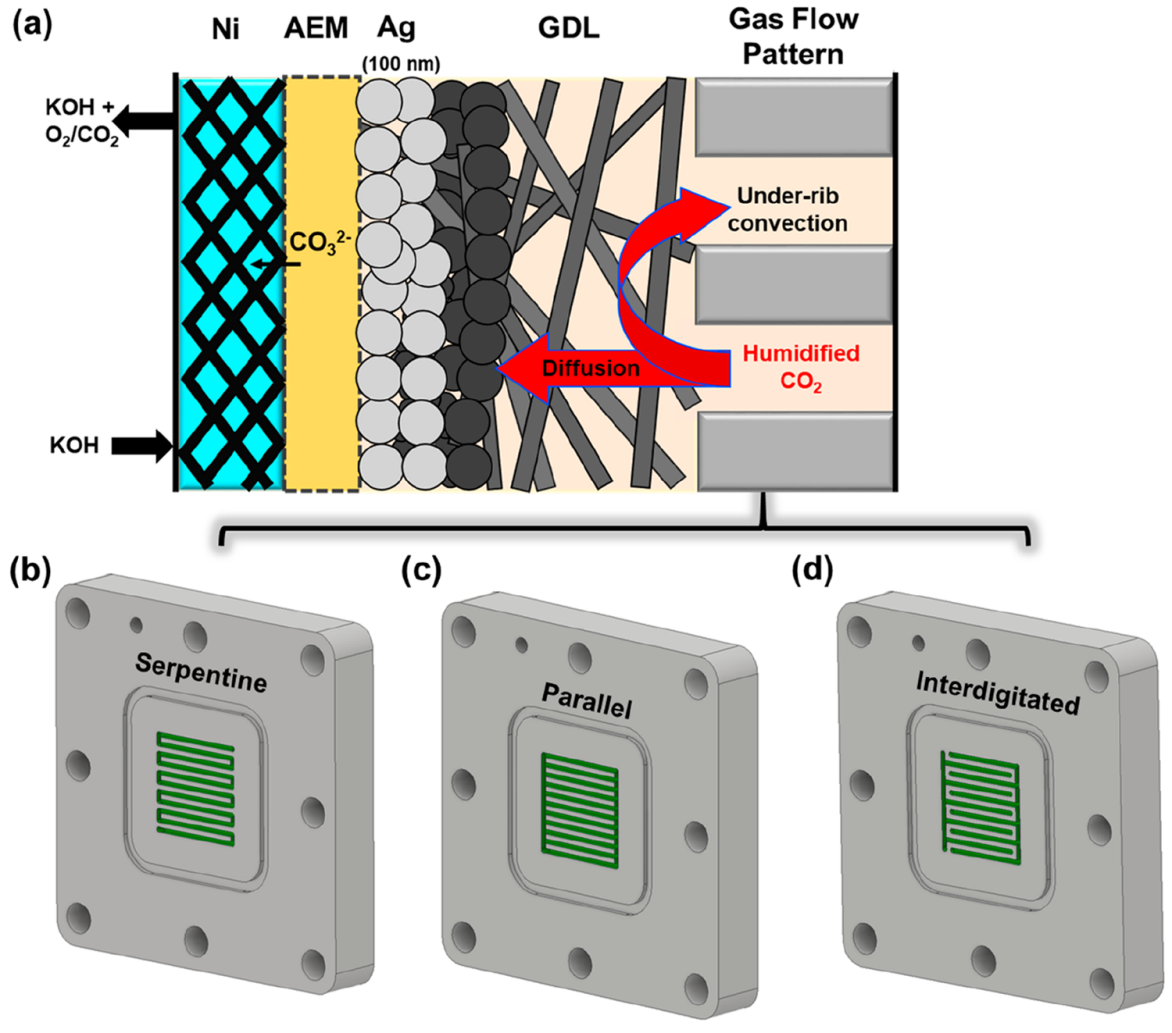
(a) Illustration of the components of an exchange MEA cell with the Ag catalyst layer sputtered on a carbon gas-diffusion layer. Shown here are the three different flow patterns at the cathode used in the study: (b) serpentine, (c) parallel, and (d) interdigitated flow patterns.

These temporal and spatial mass transport effects in CO_2_ electrolyzers lower the usefulness of the catalyst layer for CO_2_ reduction. A way of combining these mass transport effects is by considering geometric catalyst utilization of a CO_2_ electrolyzer, which can then be defined as the ratio of the planar catalyst area utilized for CO_2_RR (desired reaction) to the total planar catalyst area present in the system. Recent studies have considered broader mass transport efforts for mapping spatial electrochemical activity^22^ and engineering catalyst layers for maximizing multicarbon products from CO_2_ RR,^23−25^ but these have yet to be considered as changing in time.

One way to probe *geometric catalyst utilization* is by measuring changes in activity, selectivity, and stability using modified gas flow patterns at the cathode, which distribute reactants to the GDE. As shown in the parallel electrochemical fields of PEM electrolyzers and fuel cells, the gas flow pattern will impact mass transport and reactant distribution at the catalyst surface significantly.^26−28^ The three commonly adopted flow patterns are the serpentine, parallel, and interdigitated designs. As shown in Figure 1b, a serpentine flow channel has a single fluid flow path from the inlet to outlet of the reactor, resulting in a plug flow configuration.^29^ The transport mechanism of reactants through the GDE to the catalyst layer is a combination of diffusion and convection under the ribs (under-rib convection) driven by higher pressure drops.^30^ In contrast, a parallel flow channel exhibits a very low pressure drop^31^ due to the distribution of fluid into parallel channels (Figure 1c). Due to insignificant differences in gas pressure between each channel, through-plane and in-plane diffusion becomes the primary mode of mass transport from the gas channel to the catalyst. The final flow pattern commonly adopted is the interdigitated design where flow paths are dead ended,^32^ making the reactant gases flow through the GDE by forced convection (Figure 1d). Notably, the parallel and interdigitated designs both have multiple parallel paths to the outlet, while serpentine follows a singular gas channel. The differences in transport properties of each design are then a possible tool for assessing catalyst utilization in CO_2_RR systems if paired well with experimental and modeling findings.

In this study, we performed CO_2_ electrolysis on a Ag GDE using three different flow patterns at the cathode side of an MEA electrolyzer. The different flow patterns were employed to better understand the factors impacting geometric catalyst utilization using measured Faradaic efficiency and modeled CO_2_ concentrations. In addition, mass transport limitations due to salt precipitation were studied by imposing a PTFE blockage at the gas flow pattern, obstructing reactants from reaching catalyst sites. Consequently, the serpentine flow pattern showed the highest partial current density for CO production (205 mA/cm^2^) and the highest resistance to flooding, resulting in a higher catalyst utilization. Spatial variations in CO_2_ concentration were estimated using a 3D mass transport and fluid flow model, which revealed significant differences in the reactant distribution at the GDE surface. These findings can be used to formulate design rules for industrially relevant CO_2_ electrolyzers.

The MEA cell with a Ag GDE cathode, Ni foam anode, and anion exchange membrane (AEM) combined into one unit is shown schematically in Figure 1a. Humidified CO_2_ was fed as the reactant through the flow channel at the back of the GDE, which is then distributed to the catalyst layer by diffusion and convection through the GDL. Critically, the gas flow pattern on the flow plate at the back of GDE impacts the degree of CO_2_ transport to the entire geometric area (5.06 cm^2^), which is much larger than the channel area itself (2.53 cm^2^). Gas transport from the gas channel to the covered areas of the GDE is then needed to achieve full geometric catalyst utilization. We designed cathode end plates made of stainless steel with identical gas channel areas and similar rib spacing with serpentine, interdigitated, and parallel flow patterning. The channels differ, however, in their means of gas distribution. For each of these flow fields, we performed CO_2_ electrolysis using a Ag GDE for CO and formate production and 0.5 M KOH as anolyte in an exchange MEA configuration (see Figure S1 for details of the setup).

Two types of experiments were performed to analyze different effects related to geometric catalyst utilization. In the first set of experiments, we specifically examined product selectivity under varied current densities in low reaction times (<10 min). We can then assess CO_2_ distribution in the absence of flooding or salt precipitation. In the second set of experiments, we analyze longer experiments where flooding and salt formation are known to occur and observe the differences in performance for the different flow fields. We can then separate catalyst utilization into two time scales.

In the first set of experiments, CO_2_RR was performed at constant cell voltages ranging from −2.0 to −3.0 V in 20 min increments (Figure S2). As shown in Figure 2a,b, we found that CO_2_RR using all three flow patterns showed similar partial current densities for CO and H_2_ at lower cell voltages. At these lower current densities, we then conclude that CO_2_ can then reach all catalytic surfaces equally, and the catalyst performance is similar. However, at a higher cell voltage of −2.76 V, the serpentine flow pattern performed better, achieving a CO partial current density (*j*_CO_) of 205 mA/cm^2^. The interdigitated flow pattern also showed a similar *j*_CO_, but for the parallel flow pattern, a significant decrease in *j*_CO_ was observed, dropping to 153 mA/cm^2^. At the same time, *j*_H2_ increased to 74 mA/cm^2^ at −2.76 V for the parallel flow pattern, suggesting mass transport limitation arises. To investigate this, we built a numerical transport model of the gas flow channel and GDE similar to our previous work (Table S1).^33^

**Figure 2:**
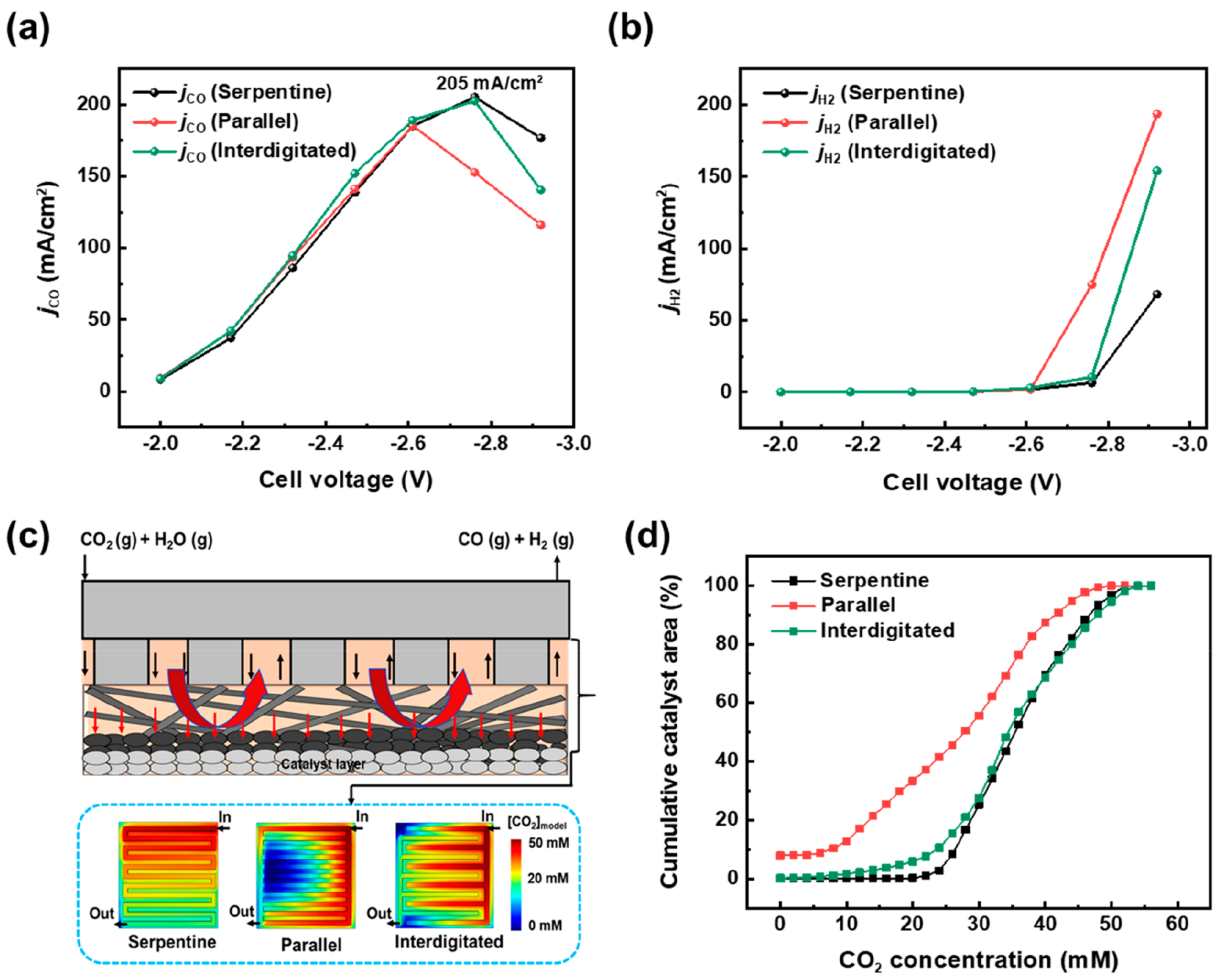
Partial current density of (a) CO and (b) H_2_ for the different flow channels. (c) Schematic of the transport model used to estimate the spatial CO_2_ distribution inside the reactor. (d) Cumulative distribution plot of catalyst area with CO_2_ access for the three flow patterns calculated from the model at a cell voltage of 2.76 V.

Observing the modeling results in Figure 2c,d, significant differences in CO_2_ distribution are predicted at the interface of the microporous layer and catalyst layer for the three flow patterns. In particular, the modeling results showed a radial distribution of CO_2_ on the GDE surface for a parallel flow pattern that forms a dead zone near the center of the GDE (Figure S3). Consequently at −2.76 V, over 8% of the GDE surface has no CO_2_ access (Figure 2d), and these regions would primarily produce H_2_ from water present at the catalyst surface. In contrast, the serpentine flow pattern shows no CO_2_ mass transport limitation and a relatively homogeneous reactant distribution (black line). The interdigitated flow pattern is closer to the serpentine channel in terms of CO_2_ distribution at the GDE surface as shown in Figure 2d, due to convection dominated transport from the gas channel through the GDE that ensures a relatively high CO_2_ concentration under the steel channel ribs. The effect of gas channel path length and under-rib convection can be seen directly in the different pressure drops between the inlet and outlet for each flow field. Here, the serpentine channel had a pressure drop 81% larger than the interdigitated channel and 936% larger than the parallel channel (see Table S2).

To further determine how catalyst utilization changes with time, we performed electrolysis at a geometric current density of 300 mA/cm^2^ for 1 h for the three flow fields. This operating range was chosen, as previous literature shows that flooding and salt precipitation will happen in a short period of time, allowing for the catalyst utilization to be assessed during a 1 h test. The advantage of performing constant current operation is that the total charge applied is fixed, which results in a constant mole of 2e^–^ products that are produced (Table S3), in this case, CO, H_2_, and formate (HCOO^–^). Although CO and H_2_ can be measured continuously, formate is only measured at the end of the test by sampling the anolyte and flooded drops from the cathode GDE. Formate was quantified using HPLC analysis (Tables S4 and S5), leading to a total FE of 90–93%. Some of the missing products can be attributed to the oxidation of formate ions at the anode as previously reported.^34^

As shown in Figure 3a,b, the serpentine flow pattern showed a relatively stable CO selectivity of 65% and a H_2_ selectivity of 2.5% during 1 h of operation. However, for the interdigitated and parallel flow patterns, drops in CO selectivity to 46.2% and 19.7% were observed, respectively, after 1 h of operation. The drop in CO selectivity and increase in H_2_ indicate that CO_2_ is increasingly unable to reach all parts of the Ag catalyst over time and that flooding or salt formation for the three patterns occurs after different periods of time. In addition, a higher formate selectivity was also observed for the parallel flow pattern (Figure 3c). This increase in formate selectivity can be attributed to a higher *H coverage at the catalyst surface due to the depleted local CO_2_ concentration as has been shown in previous reports.^35−37^

**Figure 3:**
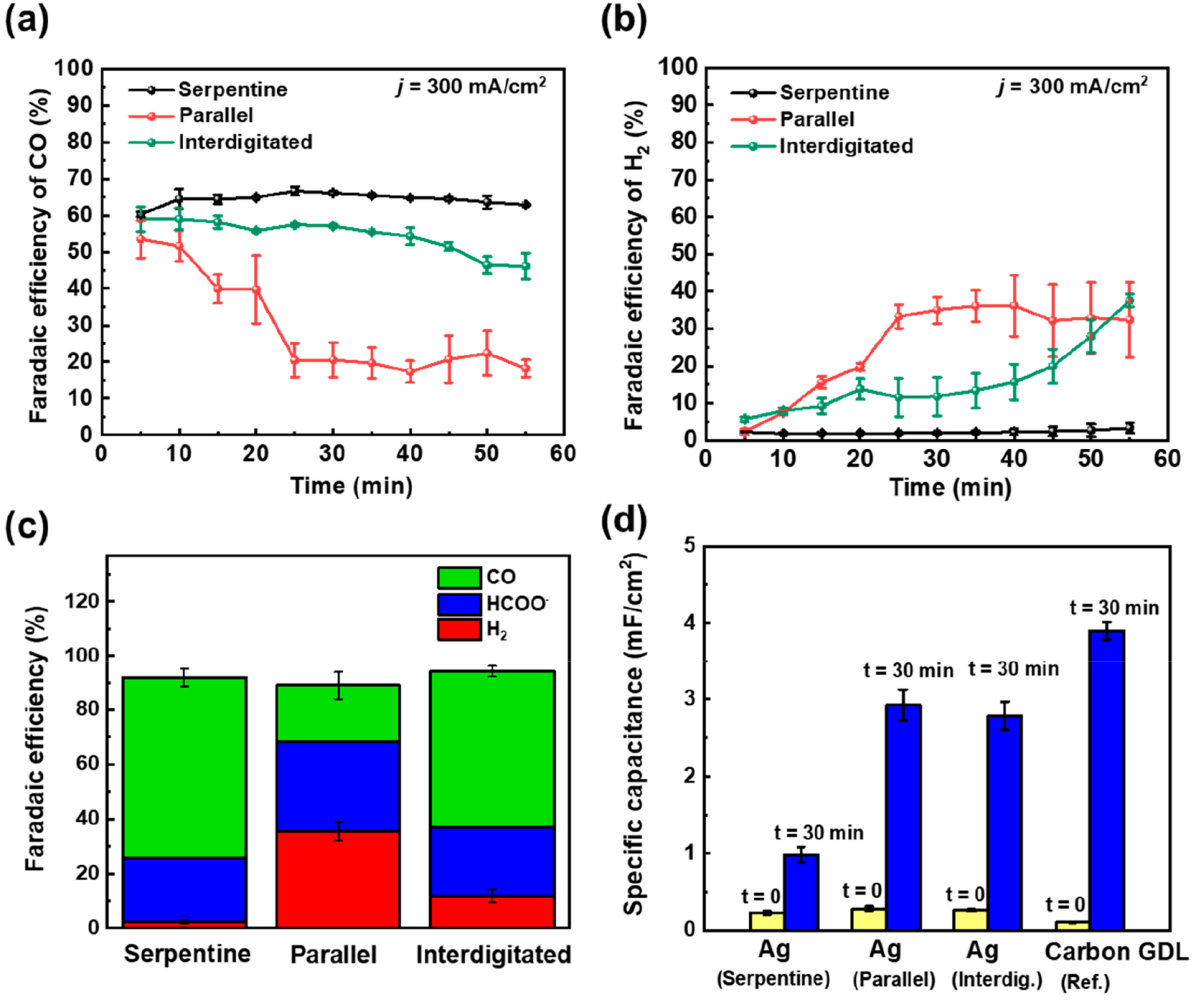
Faradaic efficiency of (a) CO and (b) H_2_ with time during 1 h of electrolysis. (c) FE of CO, H_2_, and HCOO^–^ at 300 mA/cm^2^ after 30 min of electrolysis. (d) Specific capacitance of a Ag GDE and bare carbon GDL before and after 30 min of electrolysis. Error bars represent the average of three independent experiments.

To examine the effect of flooding on catalyst utilization over time, we performed electrochemical double-layer capacitance (EDLC) measurements before and after 30 min of electrolysis (Figure S4). EDLC is a technique that can be used as a proxy for the wetted area of GDE by observing how the capacitance of a system changes over time.^38^ Since the Ag catalyst layer (100 nm sputtered silver) has a fixed surface area that is assumed to be fully wetted, increases in capacitance during operation can be attributed to the wetting of the carbon in the GDL via flooding. One can then obtain specific capacitance values by dividing measured EDLC with the geometric area of the cathode to approximate the degree of flooding of the GDL.

As shown in Figure 3d, specific capacitance values clearly reveal that different degrees of water are present in the carbon GDL when operated using different flow patterns. The serpentine flow pattern showed the lowest specific capacitance of 0.98 mF/cm^2^ after 30 min of electrolysis, suggesting a smaller wetted area of the carbon GDL and a higher resistance to flooding. On the other hand, both the parallel and interdigitated flow patterns show around a 3-fold increase in specific capacitance (2.8 mF/cm^2^) compared to that of the serpentine case. In addition, a bare carbon GDL with no Ag catalyst layer showed a specific capacitance of 3.7 mF/cm^2^ after 30 min of electrolysis. These results reveal three important findings. First, a clear increase in the fraction of flooded catalyst pores occurs for the Ag GDE when operated with parallel and interdigitated flow patterns. Flooded areas will then prevent CO_2_ from traveling from the gas channel through the GDE to all catalyst sites, thus lowering catalyst utilization and increasing HER.^39^ Second, an inverse correlation between CO selectivity and specific capacitance is observed over time showing that the *catalyst utilization* for CO_2_ electrolysis is significantly affected by the wetted area. And last, despite the interdigitated channel showing a similar degree of flooding to the parallel channel (Figure 3d), the CO performance is maintained over a much longer period.

Observing the results in Figure 3, the single-path serpentine channel clearly outperforms the two multipath channels. When we look at the salt deposition at the end of the 300 mA/cm^2^ experiments, however, all flow field patterns are heavily blocked by KHCO_3_ (Figure S5) and water (Figures S6 and S7). We then wanted to perform more controlled experiments to determine how gas channel blockages (in the form of liquids or salts) may impact catalyst utilization of an entire GDE and determine the reasons on why some flow patterns can be more resistant to changes in performance. Specifically, these control experiments should be performed with pristine GDEs and gas channels initially devoid of water or salt. To accomplish this, we placed a PTFE blockage in the gas flow channel behind the GDE from *t* = 0. We can then observe with more control the distribution of CO_2_ to different areas of the silver catalyst surface.

As shown in Figure 4a, we found that there was relatively no difference in CO selectivity for the serpentine flow pattern with or without the PTFE block. This suggests that the reactant gas can bypass such blockages due to the continuous flow path from the inlet to the outlet of the reactor while still allowing CO_2_ to reach all parts of the 5 cm^2^ catalyst area. A consequence of this, however, is that the modeling results predict a substantial increase in the inlet pressure of the reactor from 143 Pa with no PTFE block to 749 Pa with the PTFE block (Table S2). Such a large pressure drop increase indicates that CO_2_ flow can subvert the blockage by allowing for the entire flow to go in-plane through the GDE (Figure S8). These observations highlight the benefit of a single-path flow field in the event of flooding or salt formation (Figure S9) in the gas channel, at the expense of increased pressure drops. The modeling results shown in Figure 4c confirm the experimental conclusion, revealing that only a small portion of catalyst area is predicted to be without CO_2_ access (3%). Such benefits of higher pressure drop are also in agreement with a recent report on a Au GDE, where a higher pressure drop was found to increase reactant transport and stave off flooding.^40^

**Figure 4:**
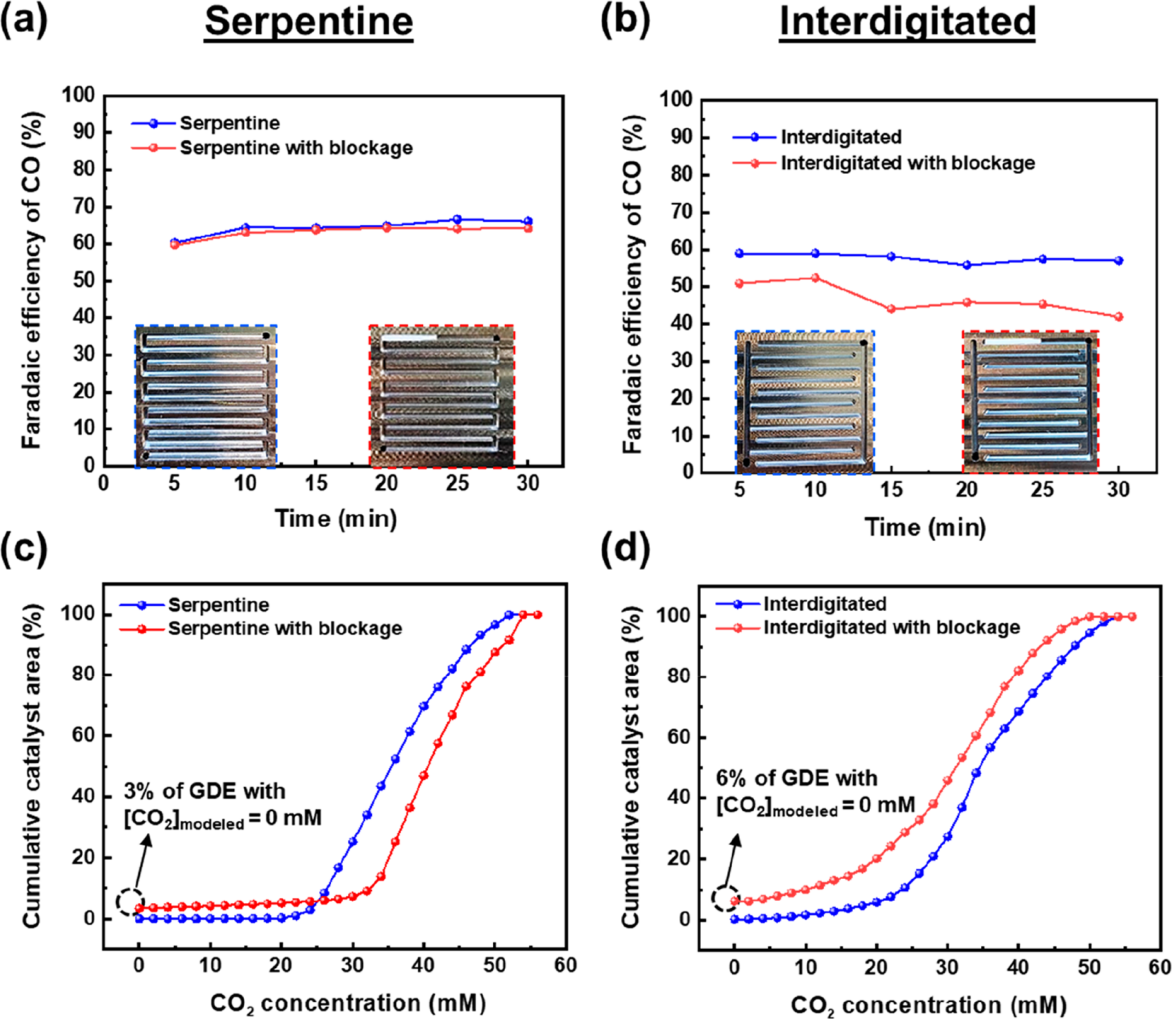
Experimental results of CO selectivity with and without the PTFE block for (a) serpentine and (b) interdigitated flow patterns. The insets depict the gas flow pattern with and without the PTFE blockage. Modeling results show the cumulative distribution plot of catalyst area with CO_2_ access with and without the PTFE blockage for (c) serpentine and (d) interdigitated flow patterns.

For the interdigitated flow pattern, a PTFE block was placed in the first set of interdigitated fingers (see Figure 4b). In contrast to the serpentine case, the interdigitated flow pattern with the PTFE block showed an 8% drop in CO selectivity compared to the unblocked case (50.9%) after 5 min. This gradually decreased to 41.9% after 30 min (Figure 4b). Further, unlike the modeling of the serpentine channel, which showed little overall difference in predicted CO_2_ distribution (Figure 4c), as much as 6.2% of the catalyst area had no access to CO_2_, which is twice as large as that observed for the serpentine case. Notably, the modeled pressure drop increase with and without the PTFE blockage was substantially less than for the single-path serpentine case. This result then elucidates the challenges with a multipath gas channel when failures begin to occur. Because gas will follow the path of least resistance, gas flow in a multipath system will avoid blocked areas as evident from the model data (Figures 4d and S7). Such observations for CO_2_RR are then not dissimilar to those in fuel cells when water is being removed.^41−44^

In summary, a single-path gas flow system is more resistant to losses in CO_2_ access, flooding, and salt precipitation as a result of higher driving pressures in the system, which allow for dispersed in-plane transport of CO_2_ through the GDE and to subsequent silver catalyst sites. When scaling up devices to larger areas, however, multipath systems will inevitably be necessary to avoid excessive pressure drops in devices. CO_2_RR research must then still look to avoid such flooding and salt formation altogether, as has been approached by a number of different strategies (pulsing, water flushing, etc.).^45,46^ Nonetheless, we hope the concepts of geometric catalyst utilization presented here bring additional thought to 2D and 3D design of CO_2_RR systems and the role that such considerations play on the observed performance across multiple time scales.

In addition, emphasis on the design parameters of the flow field patterns must be investigated to unravel the differences in CO_2_RR activity on different regions at the catalyst surface. The channel area (2.53 cm^2^), which is smaller than the surface area of GDE (5.06 cm^2^), might then have different activity due to differences in reactant concentration. For example, a higher gas flow channel-to-rib width ratio reduces under-rib convection and pressure drop, thus reducing CO_2_ flux to the catalyst surface. Optimizing such parameters might then become crucial to avoid device failures. Finally, the higher fraction of CO_2_ lost to hydroxide ions for the serpentine flow pattern (Figure S10) shows that an increase in catalyst utilization is accompanied by an overall increase in (bi)carbonate formation. This apparent contradiction is a combined result of the serpentine flow channels’ more even CO_2_ distribution throughout the entire catalyst layer (Figure 2d), which leads to overall greater carbonate formation as well as its ability to maintain a less flooded gas-diffusion layer due to under-rib convection (Figure 3d). Alternate strategies such as the use of a bipolar membrane electrode assembly^20,47^ for CO_2_ regeneration from carbonate ions might then become promising, albeit at the cost of higher cell voltages required for water dissociation in the membrane. Understanding such trade-offs might pave the way toward commercializing CO_2_ electrolyzers for industrial operation.

## Supporting Information Available

Experimental setup used for CO_2_ electrolysis, characterization of catalyst coated GDL, details of product quantification and multiphysics modeling results including all parameters used in the model (PDF)
