# Supplementary material for: Geometric Catalyst Utilization in Zero-Gap CO2 Electrolyzers
Source: ACS Energy Lett. 2022 Nov 28;8(1):222–9. doi: 10.1021/acsenergylett.2c02194 (PMC9841604; doi:10.1021/acsenergylett.2c02194)
Supplement: Supplementary file 1 — nz2c02194_si_001.pdf [file nz2c02194_si_001.pdf]

Supporting Information

Geometric Catalyst Utilization in Zero-Gap CO_2 Electrolyzers

Siddhartha Subramanian, Kailun Yang, Mengran Li, Mark Sassenburg, Maryam Abdinejad, Erdem Irtem, Joost Middelkoop and Thomas Burdyny[*]

Corresponding author email: T.E.Burdyny@tudelft.nl

Materials for Energy Conversion and Storage (MECS), Department of Chemical Engineering, Faculty of Applied Sciences, Delft University of Technology, van der Maasweg 9, 2629 HZ Delft, The Netherlands.

Experimental setup for CO_2 electrolysis

All experiments were performed in a custom made membrane electrode assembly (MEA) cell comprising of a serpentine flow channel on the anode and different flow field patterns at the cathode. Sigracet 38 BC gas diffusion layers (GDL) of 5.06 cm^2 area (2.25cm x 2.25 cm) was used as the porous transport layer. Ag catalyst layer was deposited on top of microporous layer of GDL by direct current magnetron sputtering under 3 µbar Ar flow to form a uniform film of 100 nm Ag catalyst layer. Nickel foam (3 cm x 3 cm, Recemat BV) was used as the anode. Ag GDE and Ni foam were combined with an oversized 16 cm^2 (4cm x 4cm) Sustainion anion exchange membrane (X37-50 Grade RT) to assemble the MEA. 0.5 M KOH solution was fed at the anode at a constant flow rate of 20 ml/min and recirculated using a peristaltic pump.

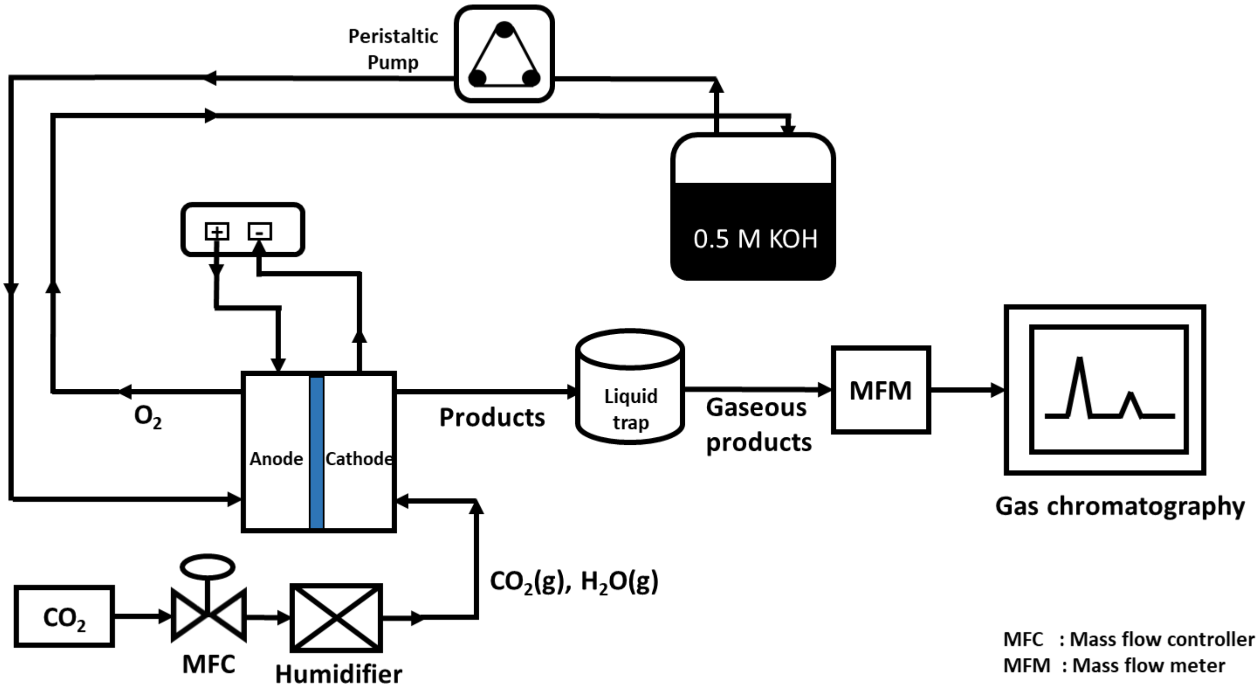

Fig. S1: Flow diagram of the experimental setup used for CO_2 electroreduction in an exchange MEA.

A constant CO_2 feed rate of 50 sccm was used and the humidity at the inlet was fixed at 75%. Electrolysis at constant cell voltages between -2.0 V and -3.0 V were performed for 20 minutes each and product quantification was performed using gas chromatography (GC) with periodic injections every 5 minutes.

Potentiostatic CO_2RR results for the three flow field patterns

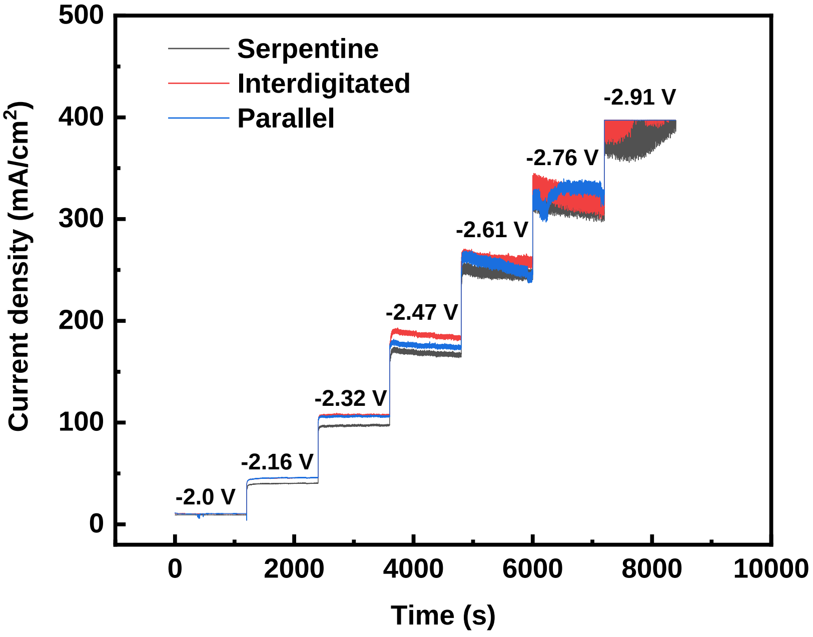

Fig. S2: Variation of current density at constant cell voltages during CO_2RR for the three flow field patterns. Constant cell voltages were held for 1200 seconds each.

Catalyst preparation and characterization

Direct current magnetron sputter deposition technique was used to sputter a thin layer of Ag catalyst layer on top of Sigracet 38 BC GDL. A thickness of 100 nm was deposited and relationship between thickness and deposition rate was calibrated by depth profiling a glass piece after 10 min of sputtering. Sputter deposition of Ag catalyst on GDL was performed at 3 µbar in an argon atmosphere at a rate of 3.125 Å/s resulting in a mass loading 0.108 mg/cm^2. The morphology of the sputtered catalyst layer was determined using scanning electron microscopy (SEM) analysis (Fig.S7).

Faradaic efficiency calculation

To estimate the Faradaic efficiency of gaseous products, the mole fractions of CO and H_2 were estimated from GC injections. The volume fraction of gas products from GC is equal to the mole fraction for ideal gases. Since the sum of mole fractions is equal to 1, the mole fraction of CO_2 exiting was calculated as,

$$x_{CO2,out} = 1 - (x_{CO} + x_{H2O} + x_{H2} + x_{H2O}) \quad \text{(S1)}$$

After calculating the mole fractions of all gaseous products, the volumetric flow rate at the outlet of the reactor measured with the MFM and used to calculate the moles of each product.

$$n_{CO} = \dot{V}_{outlet} \times x_{CO} \tag{S2}$$

$$n_{H2} = \dot{V}_{outlet} \times x_{H2} \tag{S3}$$

$$FE_{CO} = \frac{n_{CO} \times n^{e} \times F}{I} \times 100\ \% \tag{S4}$$

Here: n_{CO} - moles of CO produced, n^{e}- number of electrons involved in CO_2RR (2 for CO), F- 96485 C/mol and I - applied current (in Amperes).

Carbon balance at the cathode side

The following equations were then used to calculate the CO_2 consumption with OH^- ions by performing an overall carbon balance at the cathode side.

$$\dot{V}_{CO_2\ to\ CO} = x_{CO} \times \dot{V}_{outlet} \tag{S6}$$

$$\dot{V}_{H_2} = x_{H_2} \times \dot{V}_{outlet} \tag{S7}$$

$$\dot{V}_{residual\ CO_2} = \dot{V}_{outlet} - \left(\dot{V}_{CO_2\ to\ CO} + \dot{V}_{H_2}\right) \tag{S8}$$

$$\dot{V}_{CO_2\ to\ HCOO^-} = ((1 - x_{CO} - x_{H2}) \times \frac{j \times A}{n^{e} \times F}\ mol/s \times 22.4 \times 60 \times 1000\ )\ ml/min \tag{S9}$$

$$\dot{V}_{CO2\ to\ OH^-} = \dot{V}_{inlet} - (\dot{V}_{residual\ CO_2} + \dot{V}_{CO_2\ to\ CO} + \dot{V}_{CO_2\ to\ HCOO^-}) \tag{S10}$$

$$j_{loss} = \frac{n^{e} \times \dot{V}_{CO2\ to\ OH-} \times F}{A} \tag{S12}$$

Model description

A 3D geometry of the cathode compartment (5cm^2 area) comprising of the three flow channel designs were modelled with the same length (2.1 cm), width (1 mm) and depth (1 mm) in COMSOL Multiphysics 5.5. A carbon GDL of dimensions (2.25 cm x 2.25 cm x 0.325 cm) was modelled and placed in contact with the flow field pattern with each one consisting of 11 channels and 10 ribs. The numerical simulations were performed using a MUMPS general solver with a relative tolerance of 0.001 to calculate the CO_2 concentration gradient in the gas channel and catalyst surface.

Electrochemical reduction of CO_2 to CO was modelled and the competing hydrogen evolution reaction was not taken into account. The electrochemical reduction reaction occurring at the cathode is a 2e$^-$ reduction reaction:

$$CO2\,RR: \; CO_2 + H_2O + 2e^- \rightarrow CO + 2OH^-$$

All parameters used in the model were taken from the experimental conditions and the properties of the Sigracet 38BC GDL. The following assumptions were made in the model:

i) The system operates at steady-state conditions
ii) Carbon GDL is assumed to be isotropic with constant porosity and permeability since the in-plane diffusion is higher than the through plane diffusion
iii) Both diffusion and convection from the channel to the catalyst surface are taken into account
iv) A uniform current distribution is assumed at the catalyst surface
v) Isothermal at 298 K with no thermal diffusion gradients

Brinkmann equation and mixture diffusion model

The fluid flow in the flow channels were modelled using the Brinkmann equations which is a modified form of the Navier stokes equation for porous media flows. A no-slip boundary condition was imposed on the channel walls. A slip condition was used at the channel-GDL interface since the normal component of velocity is zero at this interface. Single phase compressible flow was assumed. An inlet boundary condition was given by a normal inflow velocity defined by the flowrate ($\dot{V}_{in}$) over inlet cross

sectional area of the channel (Dirichlet boundary condition). $\dot{V}_{in}$ was fixed at 50 sccm as used in the experiments. Constant pressure at the outlet of the cell measured from experiments was used as the boundary condition at the outlet (Neumann boundary condition) with the suppression of backflow.

The Brinkman equation solves for the velocity and pressure distribution in the GDL. It was coupled with the mixture diffusion model which takes into account of diffusion and convection through the GDL. All parameters used in the model can be found in Table S1. For the meshing, a free tetrahedral mesh with a fine mesh size was used for the channels and a swept mesh was used for the GDL (98023 domain elements, 24196 domain elements and 2894 edge elements).The velocity and pressure field in the gas channels were solved using:

$$\rho(\mathbf{u}\cdot\boldsymbol{\nabla})\mathbf{u} = \boldsymbol{\nabla}\cdot\left[-p\boldsymbol{I} + \mu(\nabla\mathbf{u} + (\nabla\mathbf{u})^{\mathrm{T}}) - \frac{2}{3}\,\mu(\nabla\cdot\mathbf{u})\mathbf{I}\right] + \mathrm{F}$$

$$\nabla\cdot(\rho\mathbf{u}) = 0$$

In the GDL, the velocity and pressure was calculated using:

$$\frac{1}{\epsilon_p}\rho(\mathbf{u}\cdot\nabla)\mathbf{u}\frac{1}{\epsilon_p} = \nabla\cdot\left[-p\mathbf{I} + \mu\frac{1}{\epsilon_p}(\nabla\mathbf{u} + (\nabla\mathbf{u}^{\mathrm{T}}) - \frac{2}{3}\mu\frac{1}{\epsilon_p}(\nabla\cdot\mathbf{u})\mathbf{I}\right] - \left(\mu\kappa^{-1} + \frac{Q_m}{\epsilon_p^2}\right)\mathbf{u} + \mathrm{F}$$

$$\nabla\cdot(\rho\mathbf{u}) = Q_m$$

In these equations,

ρ is the density of the fluid, μ is the dynamic viscosity of the fluid, p is the pressure, $\mathbf{u}$ is the velocity, F is the force term, κ is the permeability of the GDE, ϵ_p is the porosity of the GDE and Q_m is the mass source.

Mixture diffusion model

To solve for the species transport in the system, a mixture diffusion model was used. Relative humidity in the inlet stream was ignored since the humidity measured experimentally at the inlet remained constant at 75%. So, we accounted for only 2 species which are CO_2 and CO. The molar flux of the species were calculated using the following equations:

$$\nabla \cdot j_i + \rho(\mathbf{u} \cdot \nabla)\omega_i = R_i$$

$$N_j = j_i + \rho\mathbf{u}\omega_{\boldsymbol{i}}$$

$$\boldsymbol{j}_i = -(\rho D_i^m \nabla\omega_i + \rho\omega_i D_i^m \frac{\nabla M_n}{M_n})$$

$$R_i = \frac{v_i i_v}{nF} + j_{loss}$$

Here:

N is the total flux vector of species i, R_i is the reaction rate for species i, **u** is the fluid velocity, $\mathbf{j}_i$ is the relative mass flux due to molecular diffusion of species i, ω_i is the mass fraction of species i, i_v is the volumetric current density, F- Faraday's constant.

Table S1 : Parameters used in the 3D mass transport and fluid flow model

Parameter	Symbol	Value	Units	Reference
Temperature	T	298	K	This work
Reference pressure	P	1	atm	This work
Diffusivity of CO into CO_2	$D_{CO2\text{-}CO}$	1.52×10^{-5}	$m^2 s^{-1}$	[1]
Porosity of GDE	eps_gdl	0.8	$-$	[2]
Permeability of GDE	kappa_gdl	7×10^{-12}	m^2	[2]
Inlet flowrate	Q_{sccm}	50	sccm	This work
Applied current density	i_{loc}	-(3000 + j_{loss})	Am^{-2}	This work
Length of channel	*l*	2.1	cm	This work
Width of channel	*w*	1	mm	This work
Depth of channel	*h_ch*	1	mm	This work
Thickness of GDE	*dep_gdl*	325	µm	[2]
Dynamic viscosity of CO_2	*mu*	1.47×10^{-5}	Pa.s	[1]

Modelling results of CO_2 concentration at the GDE surface

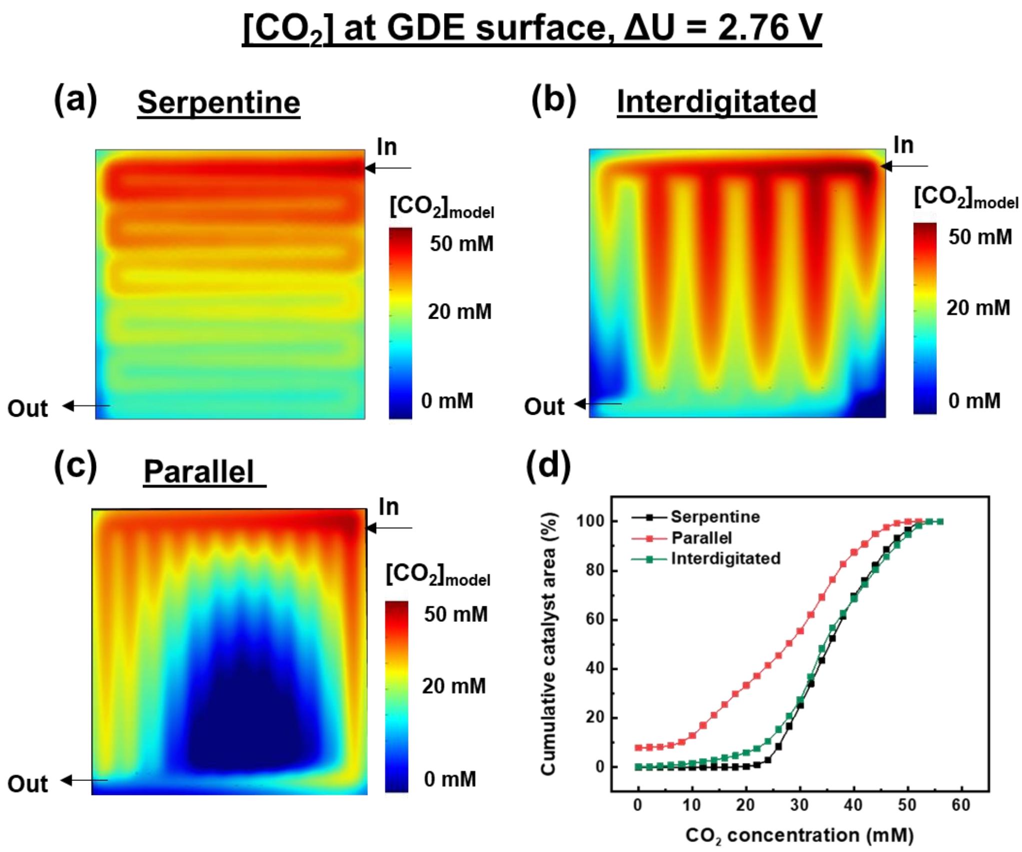

Fig. S3. Simulation results of gas phase CO_2 concentration at the interface of microporous layer and catalyst layer for (a) serpentine, (b) interdigitated and (c) parallel flow channel design. (d) A cumulative distribution plot of catalyst area with CO_2 access for the three flow patterns.

Double layer capacitance measurements

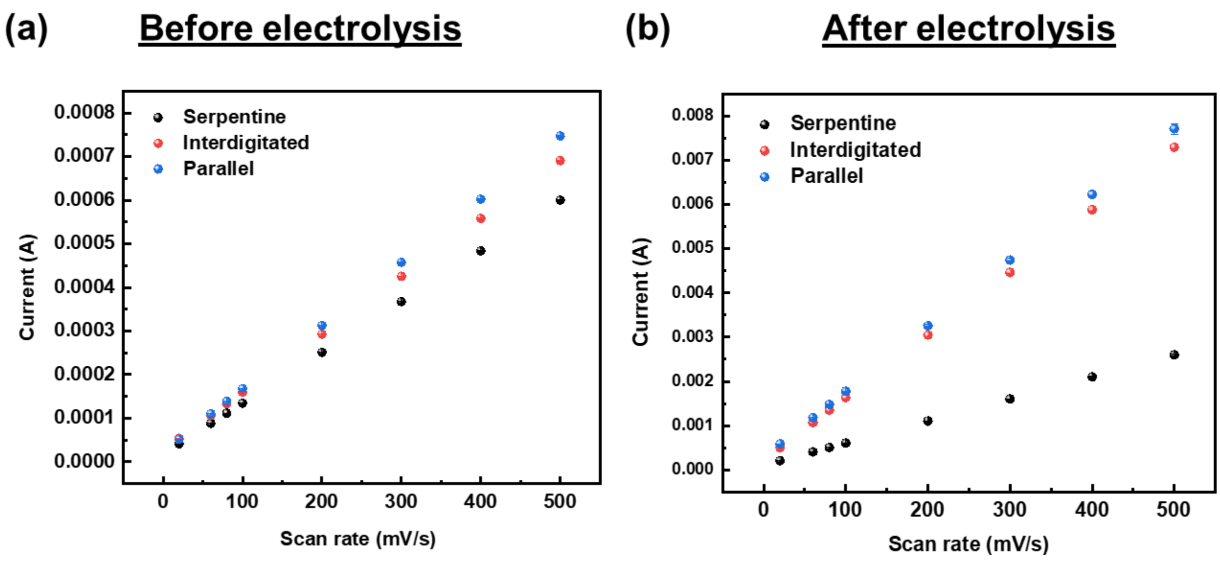

Fig. S4. Double layer capacitance of Ag sputtered GDE before and after electrolysis of 30 min at a current density of 300 mA/cm^2 for the three flow channel designs. Shown in the y-axis is the charging current averaged by anodic and cathodic currents with the scan rates on the x-axis.

Double layer capacitance measurements were performed before and after CO_2RR at 0.1 V non-Faradaic potential range and various scan rates from 500 mV/s to 20 mV/s with multiple cycles. The slope of the charging current as a function of scan rate was taken to be the capacitance.

X-ray diffraction analysis

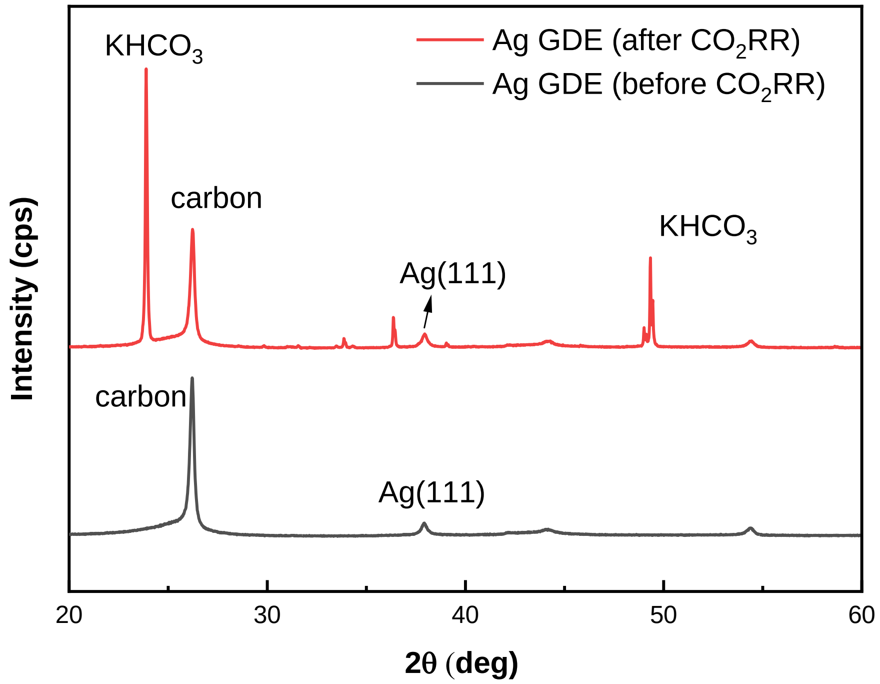

Fig. S5: XRD pattern of Ag GDE before and after CO_2 electrolysis showing the presence of Ag (111) facet. Potassium bicarbonate salt peaks are visible due to salt precipitation at the cathode.

Images of salt precipitation at the gas flow channel and GDL at 300 mA/cm^2

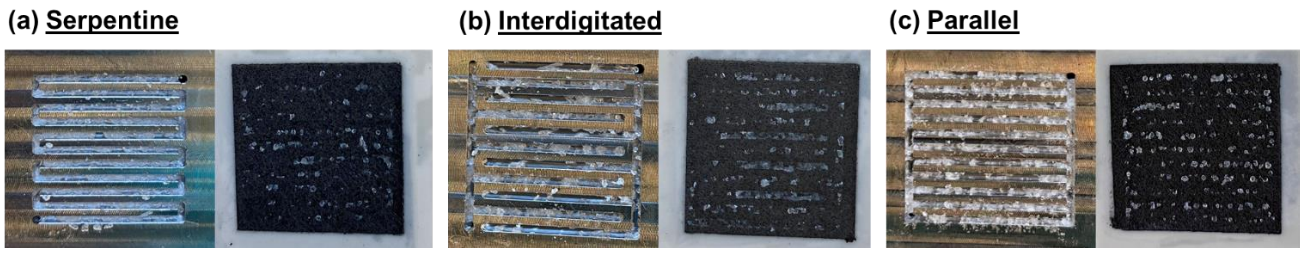

Fig. S6. Images of cathode flow channel and back of GDE after electrolysis for (a) serpentine, (b) interdigitated and (c) parallel flow channel at 300 mA/cm^2 showing salt crystals blocking the gas flow channels and the GDE.

SEM analysis

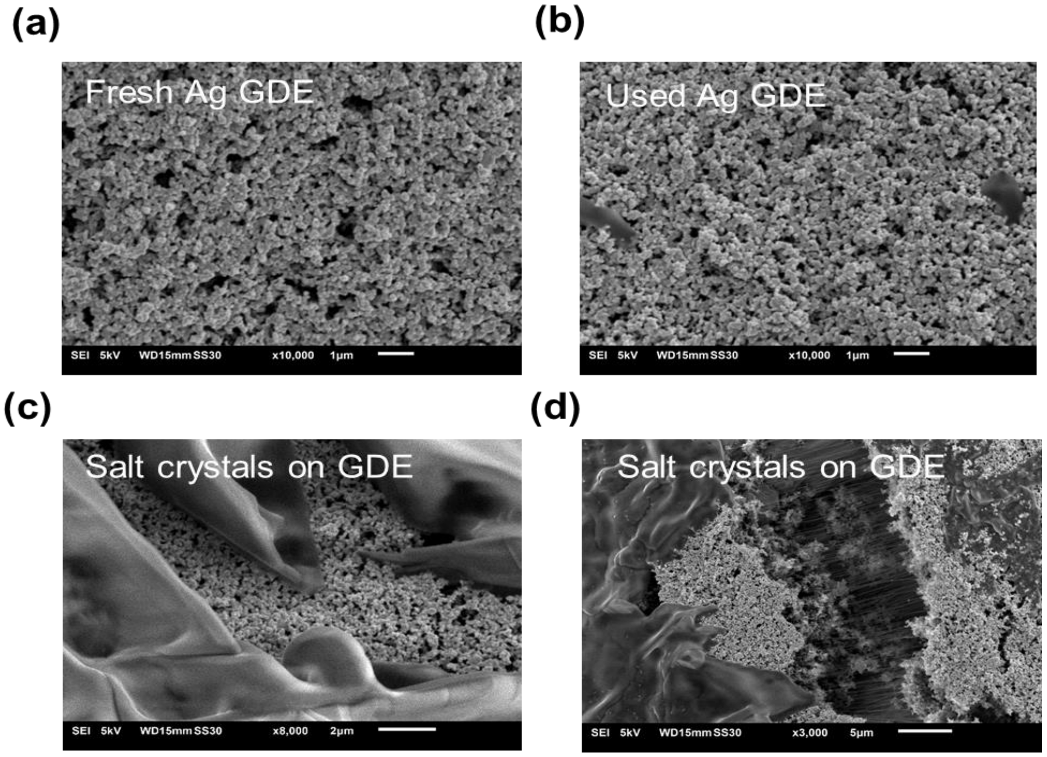

Fig. S7. SEM images of Ag catalyst sputtered on Sigracet 38 BC gas diffusion layer (a) before electrolysis, (b) after electrolysis for the serpentine flow pattern. (c) and (d) Salt crystals precipitating on the GDE surface and stretching of the carbon fibers were visible.

Table S2: Modelling results of pressure drop b/w inlet and outlet for the three flow field patterns with and without the PTFE blockage imposed at the gas channel.

Flow field pattern	ΔP b/w inlet and outlet (Pa)	Pressure drop with PTFE block (Pa)	Average velocity through GDL (mm/s)	Average velocity through GDL with PTFE blockage (mm/s)	Avg. [CO_2] at the GDE surface (mM)	Avg. [CO_2] at the GDE surface with PTFE blockage (mM)
Serpentine	143.0	749.0	7.16	14.47	38.70	36.05
Interdigitated	79.0	61.0	7.36	7.16	36.84	35.58
Parallel	13.8	15.1	0.33	0.36	30.33	26.21

Modelling flow channel design with the PTFE block

To model the experimental results of serpentine and interdigitated flow channel design with the artificially imposed PTFE blockage, we added a block of same dimensions (2 cm x 0.1 cm x 0.1 cm) in the 1st gas channel from the inlet. An interior wall boundary condition was imposed at the walls of PTFE block to the mimic blockage of gas flow.

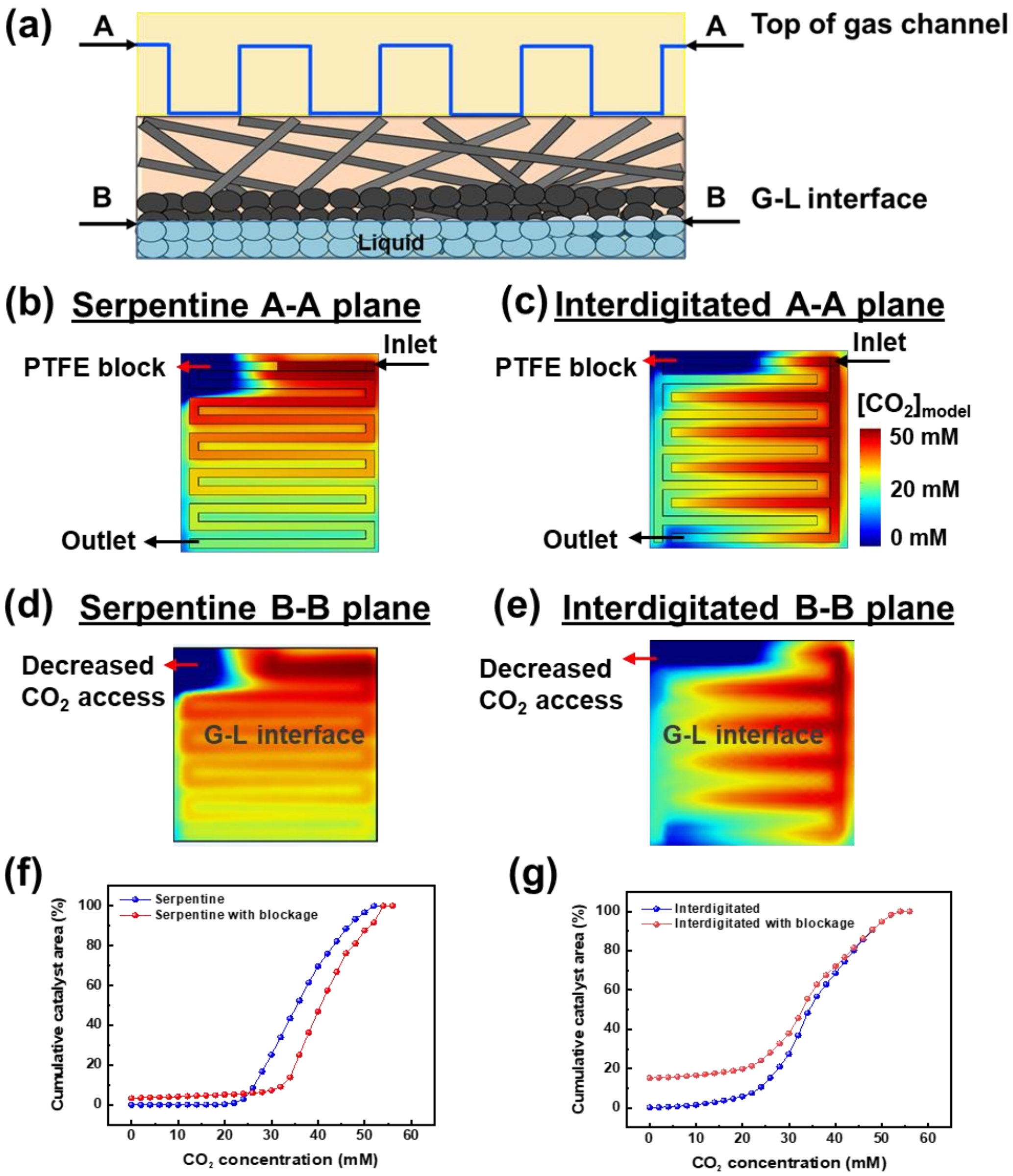

Fig. S8. (a) Schematic of the model with the imposed PTFE blockage in the gas flow channel showing two different planes, A-A plane representing top of gas flow channel and B-B plane for the gas-liquid interface. (b) CO_2 concentration from A-A plane for (b) serpentine case, (c) interdigitated case. (c) CO_2 concentration from B-B plane for (d) serpentine and (e) interdigitated case. Cumulative distribution plot of catalyst area with CO_2 access with and without PTFE block for (f) serpentine (g) interdigitated flow channel is shown.

Cross sectional SEM analysis

Cross sectional SEM analysis was performed for Ag-GDE samples after 30 minutes of electrolysis for serpentine and interdigitated flow patterns. Ag-GDEs after CO_2 electrolysis tests were cast in a mould with a polymeric resin.

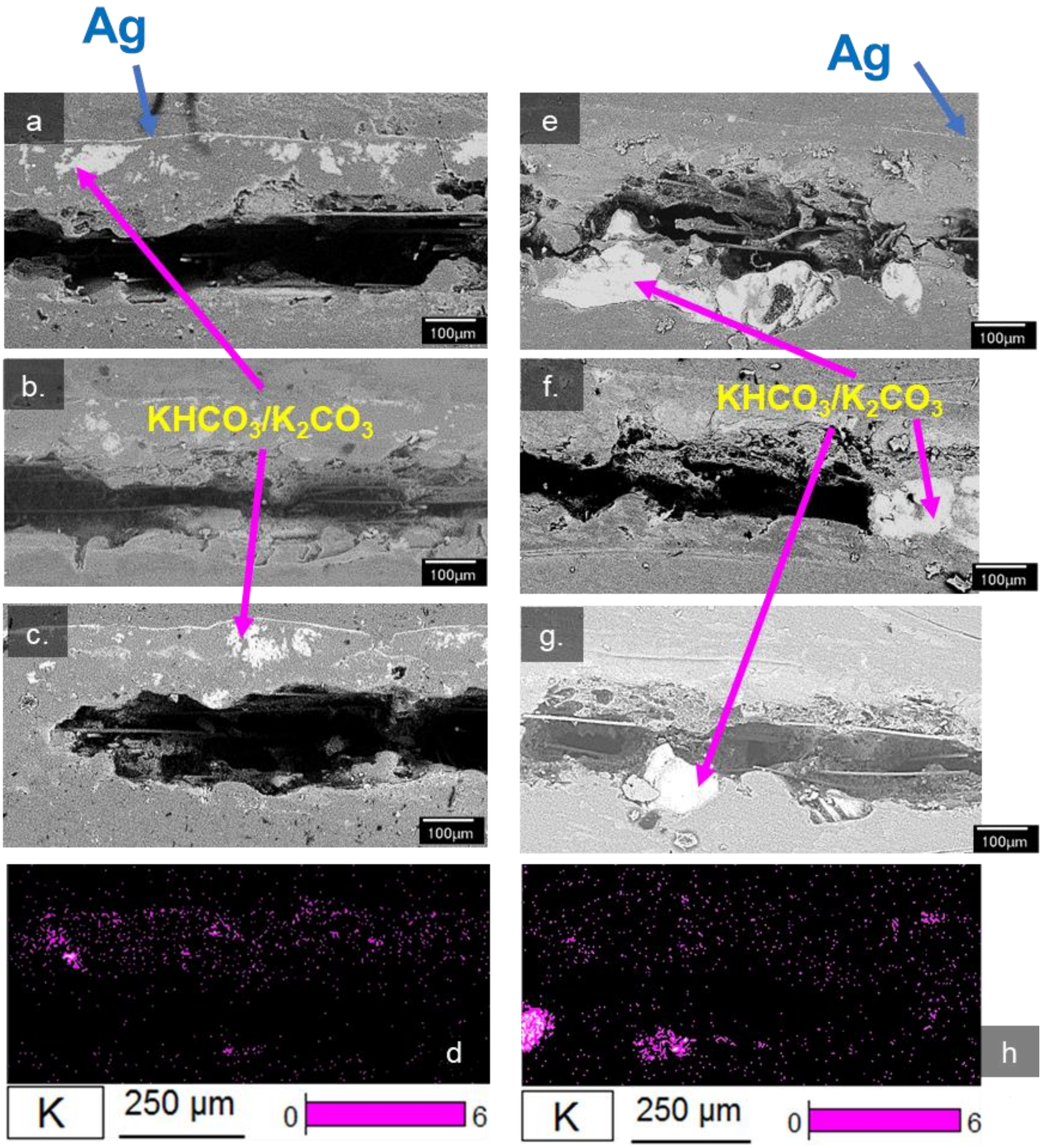

Fig. S9. Cross sectional SEM images of Ag GDE after 30 min of electrolysis for (a)-(d) serpentine and (e)-(h) interdigitated flow field patterns. Potassium salt crystals are visible in the microporous layer and carbon fiber substrate of the GDL.

After an over-night hardening step, the casts were hard polished with emery papers (500 – 2000 grid) until the mid-part of the GDEs followed with a fine-surface finishing dry pads (+4000 and diamond pads). The cross-sectional SEM analysis were conducted with a scanning electron microscope (Jeol JSM-6500F, Japan) coupled with an energy-dispersive X-ray spectrometry detector (Ultradry, Thermofischer, USA).

As shown in Fig. S9, back-scattered SEM images display a fine bright line which is the sputtered Ag catalyst layer (100 nm). The potassium (bi) carbonate salt precipitates are visible both in the microporous layer and carbon fiber layer (CFL) of the GDE (Fig S9 b,c,f,g). However, Ag-GDE operated with serpentine and interdigitated flow patterns showed differences in salt concentrations throughout the GDE. As shown in Fig S9(e), Ag-GDEs operated with interdigitated flow pattern displayed larger salt precipitates at the CFL. Although it is harder to make substantive conclusions from cross-sectional SEM and EDX analysis as shown in a recent study [4], we observe a higher concentration of K^+ ions in CFL for the interdigitated case (Fig.S9(h)). This suggests a higher degree of electrolyte intrusion (flooding) into the GDE.

In contrast, a more uniform distribution of K^+ ions are visible for the serpentine case suggesting a relatively less electrolyte intrusion (flooding) into the GDE. This agrees closely with the experimental observation of an increased rate of flooding for the interdigitated flow pattern. These differences might stem from the non-uniform CO_2 concentration at the surface of GDE for the interdigitated flow pattern as predicted from the modelling results. In addition, a higher CO_2 concentration under the ribs for the interdigitated case (Fig. S3) might explain why some regions have a higher salt precipitation. In contrast, a uniform CO_2 distribution throughout the catalyst surface might enable a more uniform salt distribution and a relatively less electrolyte intrusion into the CFL for the serpentine flow pattern.

Carbonate : hydroxide ratio at the cathode

The fraction of CO_2 reacting with OH^- ions varied for the three flow patterns at 300 mA/cm^2 constant current operation.

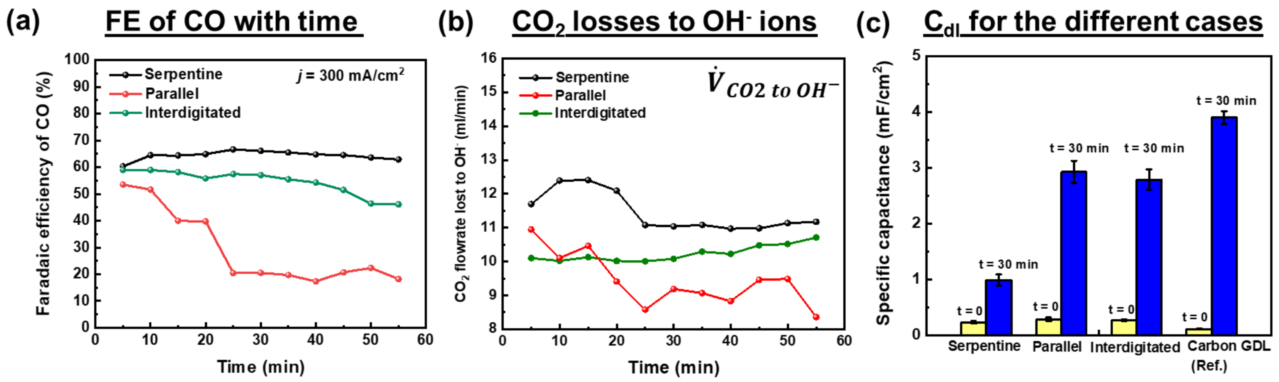

Fig. S10: (a) FE of CO with time , (b) comparison of the volumetric flowrate of CO_2 reacting with OH^- ions at 300 mA/cm^2 and (c) Double layer capacitance measurements after 30 minutes for the three flow field patterns.

As shown in Fig. S10, the serpentine flow pattern has the highest fraction of CO_2 lost to OH^- ions followed by the interdigitated and the parallel flow fields. Parallel flow pattern in particular showed a constant drop in this fraction during CO_2RR, suggesting a decrease in carbonate: hydroxide ratio at the cathode due to flooding of the GDL.

Table S3: Electrochemical and homogenous reactions occurring at the cathode of the electrolyzer.

CO_2RR to CO	$CO_2 + H_2O + 2e^- \rightarrow CO + 2OH^-$	(S13)
HER	$2H_2O + 2e^- \rightarrow H_2 + 2OH^-$	(S14)
CO_2RR to $HCOO^-$	$CO_2 + H_2O + 2e^- \rightarrow HCOO^- + OH^-$	(S15)
HCO^{3-} formation	$CO_2 + OH^- \leftrightarrow HCO_3^-$	(S16)
CO_3^{2-} formation	$HCO_3^- + OH^- \leftrightarrow CO_3^{2-}$	(S17)

Formate oxidation at the anode

Partial oxidation of $HCOO^-$ ions at the anode was measured by collecting aliquots from the anolyte every 5 min during CO_2RR at 300 mA/cm^2.

Table S4: Time dependent formate quantification from anolyte for Ag GDE using serpentine FFP.

Time (min)	$HCOO^-$ detected from anolyte (in ppm)	FE of $HCOO^-$ (%)
10	738.28	34.8
15	1069.88	33.6
20	1295.71	30.5
25	1468.69	27.6
30	1400.11	22.1

HPLC analysis

Table S5: Analysis of liquid products from CO_2RR using HPLC

Flow field pattern	$HCOO^-$ detected from anolyte (in ppm)	$HCOO^-$ detected from flooded drops in liquid trap at the cathode (in ppm)	FE of $HCOO^-$ (%)
Serpentine	740.6	558.74	23.7
Interdigitated	795.6	344.06	25.44
Parallel	1044.5	168.16	33.17

Faradaic efficiency of formate from HPLC

Sample calculation: The formate ions present in the anolyte and flooded drops in the liquid trap at the cathode were detected from HPLC measurements and the faradaic efficiency was calculated using the following equation. Here a sample calculation for the CO_2RR performed for 30 min in the serpentine flow pattern is shown. The volume of anolyte used in the experiment was 200 mL (0.5 M KOH) and the flooded drops at the cathode were diluted with DI water to make a 10 mL solution.

$$[HCOO^-]\ detected\ in\ anolyte = 740.6\ ppm = 0.7406\ gL^{-1}$$

$$Molecular\ weight\ of\ HCOO^{-} = 45\ gmol^{-1}$$

$$Volume\ of\ anolyte\ used = 0.2\ L$$

$$Moles\ of\ HCOO^{-} = \frac{0.7406\ gL^{-1}}{45\ gmol^{-1}} \times 0.2\ L = 3.29 \times 10^{-3}\ \ moles$$

$$FE_{HCOO^{-}} = \frac{Moles\ of\ HCOO^{-} \times n^{e} \times F}{I \times t} \times 100\ \% = \frac{3.29 \times 10^{-3} \times 2 \times 96485}{1.506\ A \times 1800\ s} \times 100 = 23.43\ \%$$

$$[HCOO^{-}]\ detected\ in\ liquid\ trap\ at\ cathode = 548.74\ ppm = 0.548\ gL^{-1}$$

$$Volume\ used = 0.01\ L$$

$$Moles\ of\ HCOO^{-} = \frac{0.548\ gL^{-1}}{45\ gmol^{-1}} \times 0.01\ L = 1.22 \times 10^{-4}\ \ moles$$

$$FE_{HCOO^{-}} = \frac{Moles\ of\ HCOO^{-} \times n^{e} \times F}{I \times t} \times 100\ \% = \frac{1.22 \times 10^{-4} \times 2 \times 96485}{1.506\ A \times 1800\ s} = 0.87\ \%$$

The faradaic efficiency of formate from the cathode side droplets were <1 % suggesting that most of the formate ions migrated to the anolyte through the AEM.

References

[1] E. L. Cussler, *Diffusion, mass transfer in fluid systems*, Cambridge University Press, Cambridge Cambridgeshire ; New York, 1984.

[2] *SIGRACET GDL White Paper - Fuel Cell Store*. (n.d.). Retrieved November 21, 2022, from https://www.fuelcellstore.com/spec-sheets/sigracet-gdl-white-paper-new-generation.pdf

[3] COMSOL Inc, *CFD Module User's Guide*, 2017.

[4] Kong, Y.; Hu, H.; Liu, M.; Hou, Y.; Kolivoška, V.; Vesztergom, S.; Broekmann, P. Visualisation and Quantification of Flooding Phenomena in Gas Diffusion Electrodes Used for Electrochemical CO2 Reduction: A Combined EDX/ICP–MS Approach. *Journal of Catalysis* **2022**, *408*, 1–8.
